# A Longitudinal Study of Disability, Cognition and Gray Matter Atrophy in Early Multiple Sclerosis Patients According to Evidence of Disease Activity

**DOI:** 10.1371/journal.pone.0135974

**Published:** 2015-08-17

**Authors:** Gro O. Nygaard, Elisabeth G. Celius, Sigrid A. de Rodez Benavent, Piotr Sowa, Marte W. Gustavsen, Anders M. Fjell, Nils I. Landrø, Kristine B. Walhovd, Hanne F. Harbo

**Affiliations:** 1 Department of Neurology, Oslo University Hospital and Institute of Clinical Medicine, University of Oslo, Oslo, Norway; 2 Department of Ophthalmology, Oslo University Hospital and Institute of Clinical Medicine, University of Oslo, Oslo, Norway; 3 Department of Radiology, Oslo University Hospital and Institute of Clinical Medicine, University of Oslo, Oslo, Norway; 4 Department of Psychology, University of Oslo, Oslo, Norway; University of Düsseldorf, GERMANY

## Abstract

New treatment options may make “no evidence of disease activity” (NEDA: no relapses or disability progression and no new/enlarging MRI lesions, as opposed to “evidence of disease activity” (EDA) with at least one of the former), an achievable goal in relapsing-remitting multiple sclerosis (RRMS). The objective of the present study was to determine whether early RRMS patients with EDA at one-year follow-up had different disability, cognition, treatment and gray matter (GM) atrophy rates from NEDA patients and healthy controls (HC). RRMS patients (mean age 34 years, mean disease duration 2.2 years) were examined at baseline and one-year follow-up with neurological (n = 72), neuropsychological (n = 56) and structural MRI (n = 57) examinations. Matched HC (n = 61) were retested after three years. EDA was found in 46% of RRMS patients at follow-up. EDA patients used more first line and less second line disease modifying treatment than NEDA (p = 0.004). While the patients groups had similar disability levels at baseline, they differed in disability at follow-up (p = 0.010); EDA patients progressed (EDSS: 1.8–2.2, p = 0.010), while NEDA patients improved (EDSS: 2.0–1.7, p<0.001). Cognitive function was stable in both patient groups. Subcortical GM atrophy rates were higher in EDA patients than HC (p<0.001). These results support the relevance of NEDA as outcome in RRMS and indicate that pathological neurodegeneration in RRMS mainly occur in patients with evidence of disease activity.

## Introduction

With the emergence of new disease modifying treatment (DMT) options, “disease activity free status” [1,2] or “no evidence of disease activity” (NEDA) [3] has been introduced as an ambitious goal of multiple sclerosis (MS) therapy. The rationale for this concept is that MS treatment should aim for no signs of disease activity; neither new relapses, disability progression nor new/enlarging white matter (WM) lesions.

NEDA was first introduced as an outcome measure in post-hoc studies of DMTs in clinical trials [2,4,5]. It has revealed differences between treatments and placebo [2,4,5], and sustained remission of active RRMS treated with high-dose immunosuppressive therapy and autologous hematopoietic cell transplantation (HDIT/HCT) [6]. A recent population-based cohort study found that almost half of the patients fulfilled the NEDA criteria after one year, and that NEDA status at two years predicted stability of disability the following five years better than any of the individual measures alone [3].

A shortcoming of NEDA has been the lack of knowledge concerning its relation to cognitive decline and gray matter (GM) atrophy [1]. Cognitive decline is important in MS because it is frequent and affects work and social activities of the patients [7]. Pathological studies have found that GM atrophy in MS is associated with demyelination and neuronal, axonal and synaptic loss [8,9]. Clinically this neurodegeneration is associated with both cognitive and neurological disability, even early in the disease course [10]. Moreover, early brain atrophy is associated with long term disability [11]. It is therefore highly relevant to elucidate whether cognition and GM atrophy rates are different between early MS patients with evidence of disease activity (EDA: a new relapse, disability progression or any new/enlarging WM lesion on MRI), patients without evidence of disease activity (NEDA: neither of the former) and healthy controls (HC).

This cohort study describes the disease development and GM changes after one year in a population-based collection of early relapsing-remitting MS (RRMS) patients. The patients were recruited from Oslo, Norway, where most RRMS patients are treated in the public health care system and receive standardized individualized treatment [12]. To expand current knowledge, we hypothesized that EDA and NEDA patients and HC could be separated according to disease modifying treatment (DMT), disease characteristics (EDSS), cognition (processing speed, verbal and visuospatial memory) and GM (cortical and subcortical) atrophy rates at one year follow-up.

## Materials and Methods

### Patients and controls

**Fig 1:**
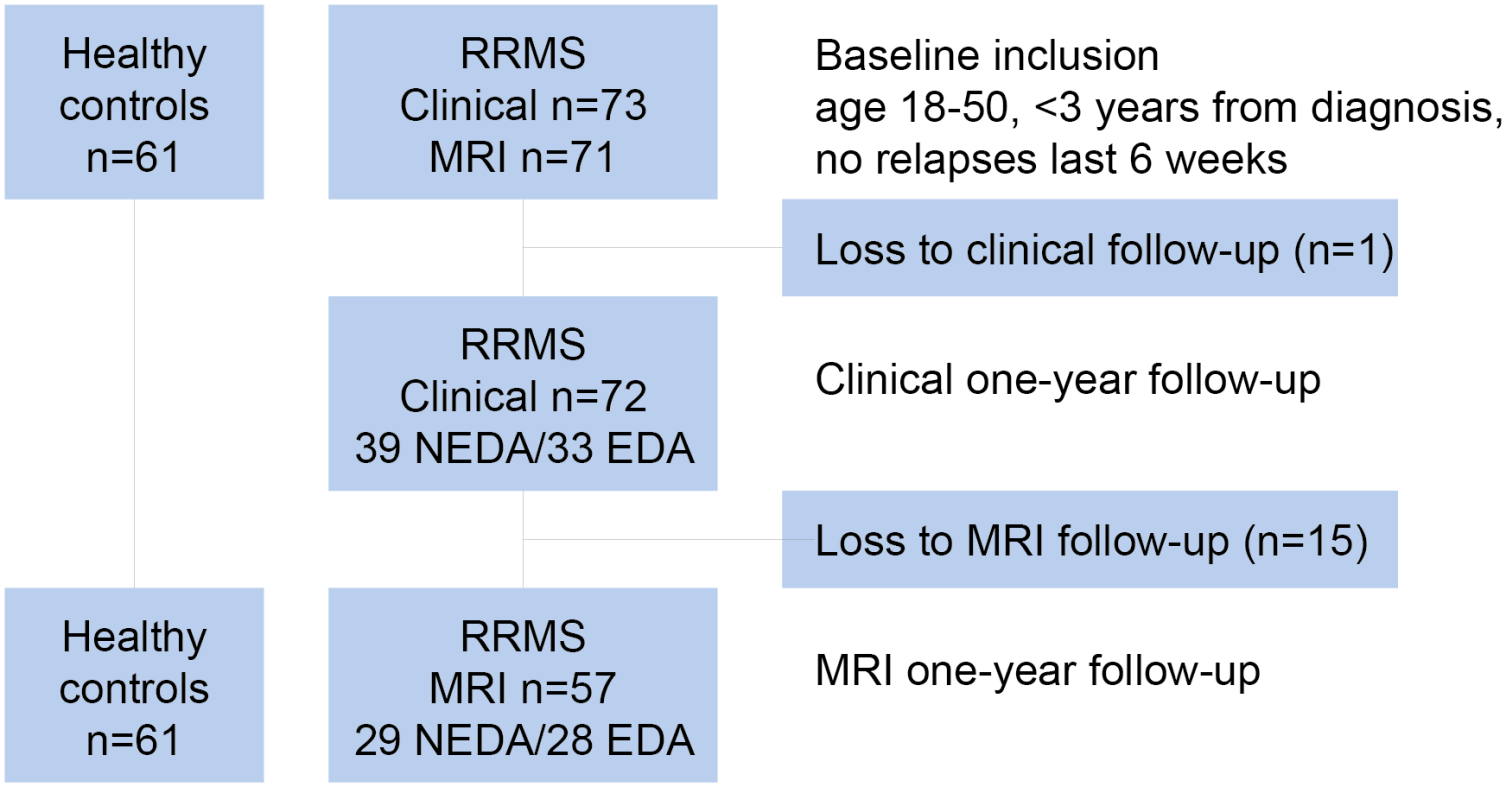
Flow chart of patients and controls. Loss to clinical follow-up: moved (n = 1). Loss to MRI follow-up: pregnancy (n = 4), no contact/declined to participate (n = 3), moved (n = 2), incomplete MRI (n = 5).

Patients diagnosed with RRMS according to the revised McDonald Criteria [13] at Oslo University Hospital, Ullevål, in the period from January 2009 to October 2012 were included. Mean time since diagnosis was 1.2 years (SD 0.8) when included in the baseline analyses in 2012. They were re-examined on average 13 months (SD 2.3) later. Baseline results including detailed MRI analyses of cortical atrophy in this cohort are published previously [14]. Details concerning inclusion and follow-up are shown in Fig 1. Patient inclusion criteria at baseline were age 18–50 and fluency in Norwegian. Exclusion criteria at baseline were other neurological or psychiatric diseases, drug abuse, head trauma, pregnancy or previous adverse Gadolinium reaction. Clinical information from 72 patients, cognitive data from 56 patients and MRI data from 57 patients were available from both baseline and at follow-up. The patients with cognitive and MRI data were mostly overlapping; for 52 patients MRI data and cognitive data were available at both time points, while at follow-up four patients had cognitive data only and five patients had MRI data only.

At follow-up 54% (39/72) of the patients were classified as NEDA. Correspondingly, 46% (33/72) showed evidence of disease activity.

Treatment decisions for the included patients were made by their neurologists independently of this study, according to the Norwegian Guidelines for MS treatment [12]. Available first line DMTs were interferons and glatiramer acetate and second line DMTs were natalizumab and fingolimod. The treatment was reconsidered with appearance of new relapses, neurological worsening, MRI progression, adverse side effects, neutralizing antibodies or for personal reasons. We applied an intention-to-treat approach in the analyses and thus reported the baseline treatment of the patients with EDA or NEDA at follow-up.

The controls were selected from the ongoing project “Cognition and plasticity through the lifespan” at the Department of Psychology, University of Oslo, from a pool of approximately 150 eligible participants [15]. HC inclusion criteria were fluency in Norwegian, no known neurological or psychiatric disease, drug abuse, head trauma, depressive symptoms (BDI>16) or subjective worries concerning cognitive function. They were matched with the RRMS patients on group level at baseline, based on age, gender and availability of MRI at baseline and follow-up. The controls were followed up after 42 months (SD 4.5).

All participants gave written informed consent and the study was approved by the regional ethical committee of South Eastern Norway (REK).

### Definition of relevant terms

*A relapse* was defined as any new neurological symptoms, not associated with fever or infection, lasting for at least 24 hours and accompanied by new neurological signs. *Disability progression* was defined as an increase in EDSS≥1 compared to baseline in the absence of a relapse the last six weeks before examination. *Radiological progression* was defined as at least one new/enlarging T2 or FLAIR WML (with or without gadolinium enhancement on T1) compared to MRI at baseline. Patients with *either* a relapse, *or* disability progression *or* radiological progression were classified as *EDA*. Patients with no relapses, no disability progression and no radiological progression at follow-up compared to baseline were classified as *NEDA*.

### Neurological and neuropsychological examination

Most patients (n = 64) underwent a full neurological examination, including EDSS, by one of two Neurostatus certified medical doctors (http://www.neurostatus.net/), within the same week as their MRI examination at baseline and follow-up. For the remaining patients (n = 8) information was collected from the patients’ medical records. Information about DMTs was collected both from patient interviews and from medical records.

At both time points, patients were tested for verbal memory (California Verbal Learning Test 2 (CVLT 2), alternate form used at follow-up [16]), processing speed (the Symbol Digits Modalities Test (SDMT) [17]) and visuospatial memory (Brief Visuospatial Memory Test—Revised (BVMT-R) alternate form used at follow-up [18]). They further filled out questionnaires on fatigue (Fatigue Severity Scale, FSS [19]) and depressive symptoms (Beck Depressive Inventory II, BDI [20]).

At baseline the patients also underwent the tests included in the Multiple Sclerosis Functional Composite (MSFC) [21], and hence performed the Paced Auditory Serial Attention Test (PASAT), the nine hole peg-test (9HP) and the timed 25 foot walk test (T25FW). The raw scores of the neuropsychological tests were used in the analyses.

### Image acquisition

Patients and controls underwent cerebral MRI examinations using the same 1.5 T Siemens Avanto scanner (Siemens Medical Solutions) with a 12 channel head coil. The controls were scanned between June 2007 and December 2008 at baseline, and between January 2011 and June 2013 at follow-up. The patients were scanned between January 2012 and January 2013 at baseline and between April 2013 and February 2014 at follow-up. The patients were instructed to lie in a standardized position in the scanner. The MRI sequence used for volumetric analyses were 3 dimensional T1-weighted Magnetization Prepared Rapid Gradient Echo (MP-RAGE) sequences, with the following sequence parameters: repetition time / echo time / time to inversion / flip angle = 2400 ms / 3.61 ms / 1000 ms / 8°, matrix 192 × 192, field of view = 240. Each scan lasted 7 min 42 s and consisted of 160 sagittal slices with a voxel size of 1.20 × 1.25 × 1.25 mm. The sequences were kept identical between the scanning periods. For clinical radiological evaluation of the patients FLAIR, T2 and pre- and post-gadolinium T1 MP-RAGE sequences were used. Details concerning the remaining sequences have been described earlier [14].

### Image analyses

Information about radiological progression was extracted from the routinely reported evaluations of the cerebral MRI scans made by neuroradiologists at the hospital, and categorized as either radiological progression or no radiological progression by the first author (GON).

The original scans from both time points were visually inspected to assure good quality before the segmentation. For volumetric analyses, the baseline images were reprocessed, so that both baseline and follow-up images of patients and controls were processed with the same software version, Freesurfer version 5.3 (http://surfer.nmr.mgh.harvard.edu). To extract reliable volume estimates, images were automatically processed with the longitudinal stream in Freesurfer [22]. The processing steps included registration of the scans to a common atlas, ensuring that minor differences in head positioning in the scanner would not affect the results. An unbiased within-subject template was created using robust, inverse consistent registration [23]. Several processing steps, such as skull stripping, Talairach transformations, atlas registration, spherical surface maps and segmentations were then initialized with common information from the within-subject template, in order to increase reliability and statistical power [22]. After segmentation, one scan was discarded because of obvious segmentation mistakes. Data from the further scans were transferred to SPSS for statistical analyses.

### Statistical analyses

We used IBM SPSS Statistics v 22 (SPSS, Chicago, IL) for statistical analyses. We visually inspected histograms and Q-Q plots of the data to assess whether the data was normally distributed. All data satisfied this normality check. We then tested for difference between patients and controls, between the patients at different time points and between subgroups (EDA, NEDA and HC) with independent samples t-tests, paired samples t-tests, χ^2^ —tests and one-way between-group analyses of variance (ANOVAs) with Bonferroni-corrected post-hoc tests as appropriate. The χ^2^ -tests performed on categorical variables with two values only (e.g. gender) were corrected for possible overestimation with Yate’s continuity correction. All results are reported based on a significance level of α = 0.05.

To control for differences in age and gender between the patient groups (EDA and NEDA) we also performed one-way between-group analyses of covariance (ANCOVAs) where appropriate, with the dependent variable of interest (volumetric measurements and atrophy rates), group as a fixed factor, and age and gender as covariates.

The scan interval was longer for HC than patients, therefore annual percent change of the cortical and subcortical volumes were estimated as described in Freesurfer version 5.3 [23]. We calculated the symmetrized annual percent change as this rate: ((follow-up-volume ÷ baseline-volume) / (time between scans) divided by the average volume (0.5 x (baseline-volume + follow-up-volume)), taking into account both the different scanning intervals and possible image differences arising from movements or MRI distortions.

## Results

### Demographics and clinical characteristics

The patients and controls included at baseline were similar concerning gender, (Table 1) as described previously [14]. There was a non-significant age difference between patients and controls included in the MRI analyses at baseline (patients 34.6 years, controls 33.5 years, t = 0.759, p = 0.449). However, because of the longer scan interval of the controls, the age of the participants at the mid-time between the two MRI acquisitions were similar in the two groups (patients 35.2 years, controls 35.3 years, t = -0.054, p = 0.957). The atrophy measurements therefore span similar age levels in both groups. The controls have one year more of education than the patients (ANOVA F(2, 133) = 3.0, p = 0.054, post-hoc Bonferroni-corrected tests revealed no significant pair-wise differences). In a previous publication we have shown that the general ability levels of patients and controls were similar, assessed with tests of vocabulary and matrix reasoning, and we therefore considered the groups suitable for comparison [14]. The moderate loss of patients to structural MRI did not alter the demographic or disease characteristics of the patient sample (S1 and S2 Tables). Therefore, we considered the patients with structural MRI data representative of the total patient cohort in the further analyses.

**Table 1:** Baseline information and follow-up time of patients and healthy controls.

	RRMS	EDA	NEDA	HC
	n = 72	n = 33	n = 39	n = 61
**Female, n (%)**	52 (72)	17 (52)^1^	35 (90)	47 (77)
**Age, years, mean (SD)**	34.3 (7.0)	33.8 (6.6)	34.7 (7.4)	33.5 (8.4)
**Education, years, mean (SD)**	15.1 (2.3)	15.0 (2.4)	15.1 (2.1)	16.1 (2.5)
**Follow-up period, months, mean (SD)**	13.4 (2.3)	13.5 (2.0)	13.4 (2.5)	41.7 (4.5)^2^

Independent samples t-tests, paired samples t-test, χ^2^ -tests and ANOVAs with Bonferroni-corrected post-hoc tests as appropriate.

p-values<0.05 indicated with:

^1^ proportion of female was different in the EDA group compared to NEDA and HC,

^2^ The follow-up period was longer in the HC group compared to the EDA and NEDA groups.

### Evidence of disease activity

**Fig 2:**
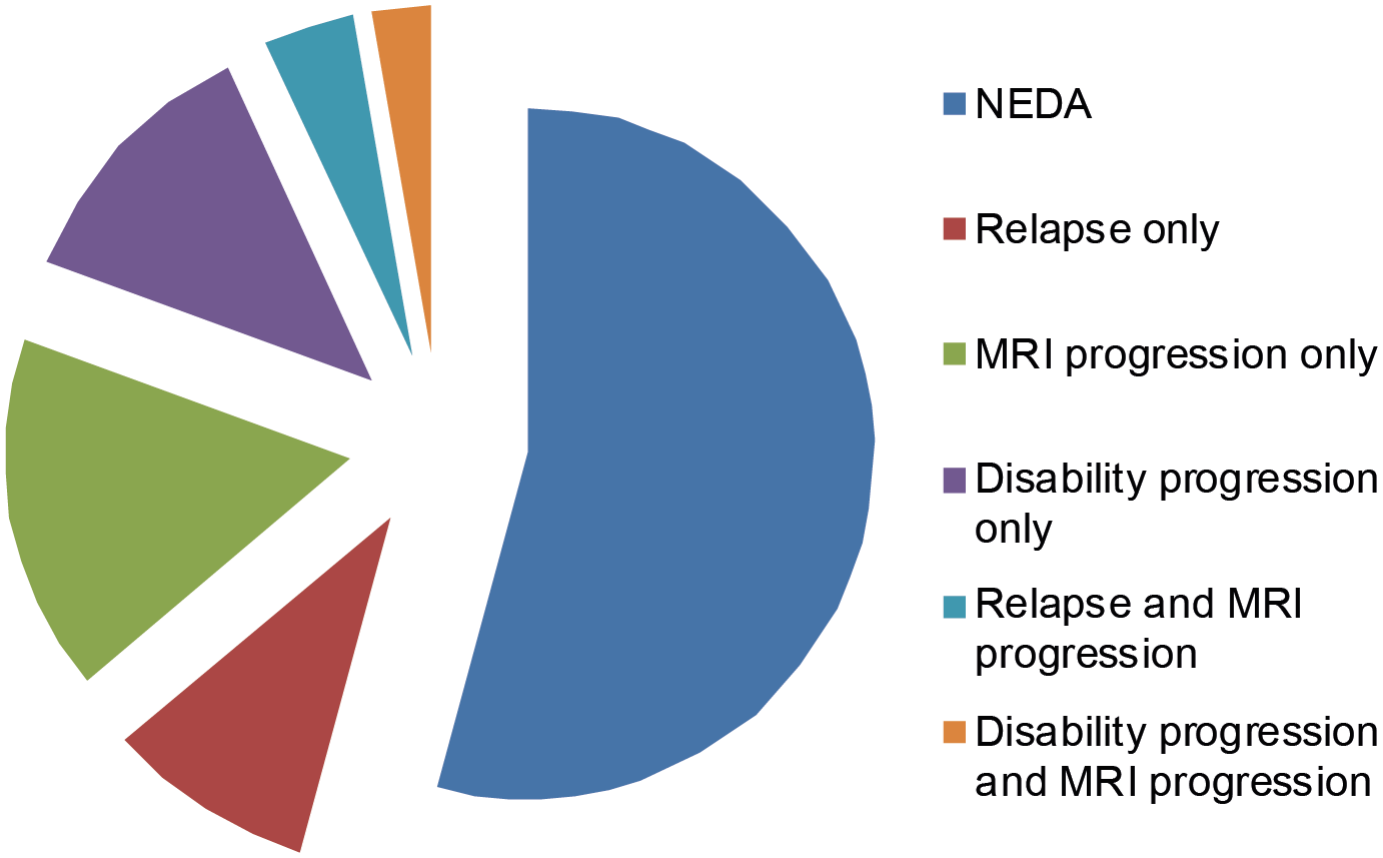
Evidence of disease activity at one-year follow-up. 54% of the patients were classified as NEDA after one year, while 46% of the patients showed either one or more evidences of disease activity.

Of the total RRMS sample, 54% (39/72) were classified as NEDA after one year. Correspondingly, 46% (33/72) showed either one or more evidences of disease activity (Fig 1). During the follow-up period 14% (10/72) experienced relapses. The mean disability level in the patient group was stable. However, 15% (11/72) showed disability progression with an EDSS increase ≥1. Radiological progression was found in 27% (17/62) of the patients compared to baseline MRI. The proportion of patients with different types of evidence of disease activity is illustrated in Fig 2.

**Fig 3:**
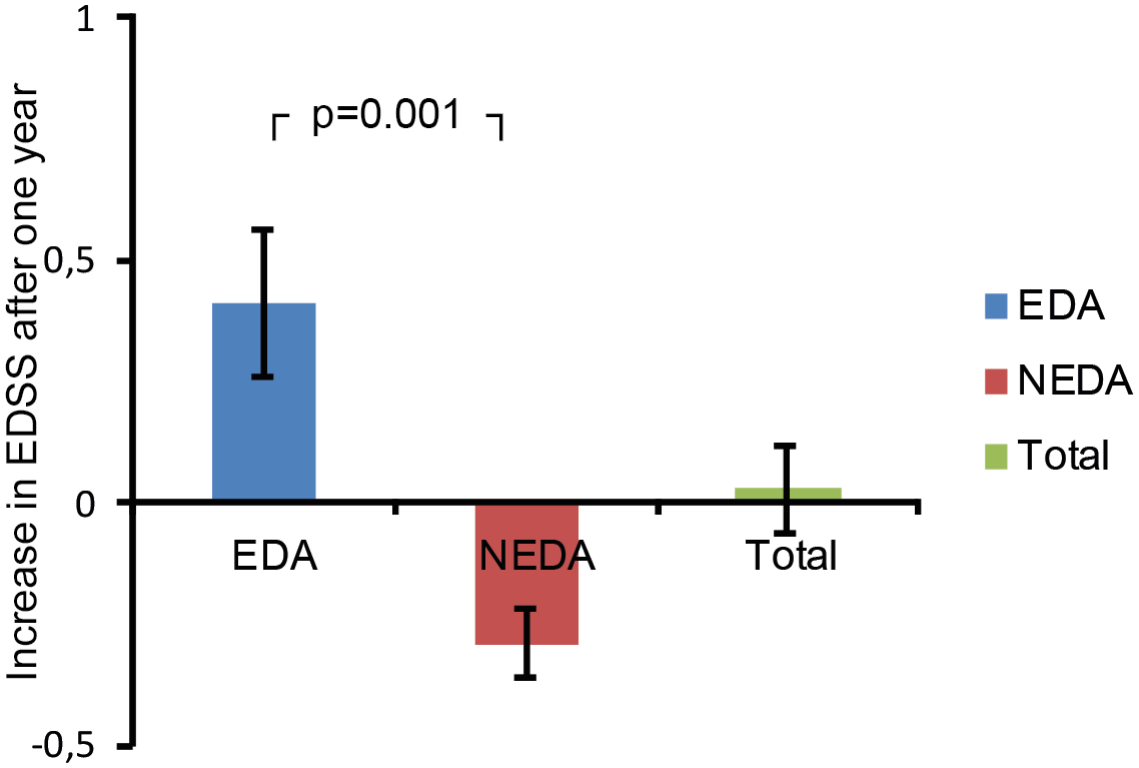
Change in disability after one year. The patient group as a whole had stable disability scores from baseline to follow-up. NEDA patients improved in disability, while the EDA patients showed a disability progression at one-year follow-up.

There were more males in the EDA group compared to the NEDA group (48% (16/33) versus 10% (4/39), p = 0.001). Age and years of education were similar (Table 1). The two groups had similar disability, disease duration and relapse rate at baseline. At follow-up, however, the disability level differed between the patient groups (p = 0.010). The change in disability during the one-year observation thus differed significantly between the groups (p = 0.001). The EDA group showed a disability progression (EDSS: 1.8–2.2, p = 0.01), while the NEDA group showed a significant improvement in disability from baseline (EDSS: 2.0–1.7, p<0.001) (Table 2a, Fig 3).

### Evidence of disease activity in different treatment groups

**Fig 4:**
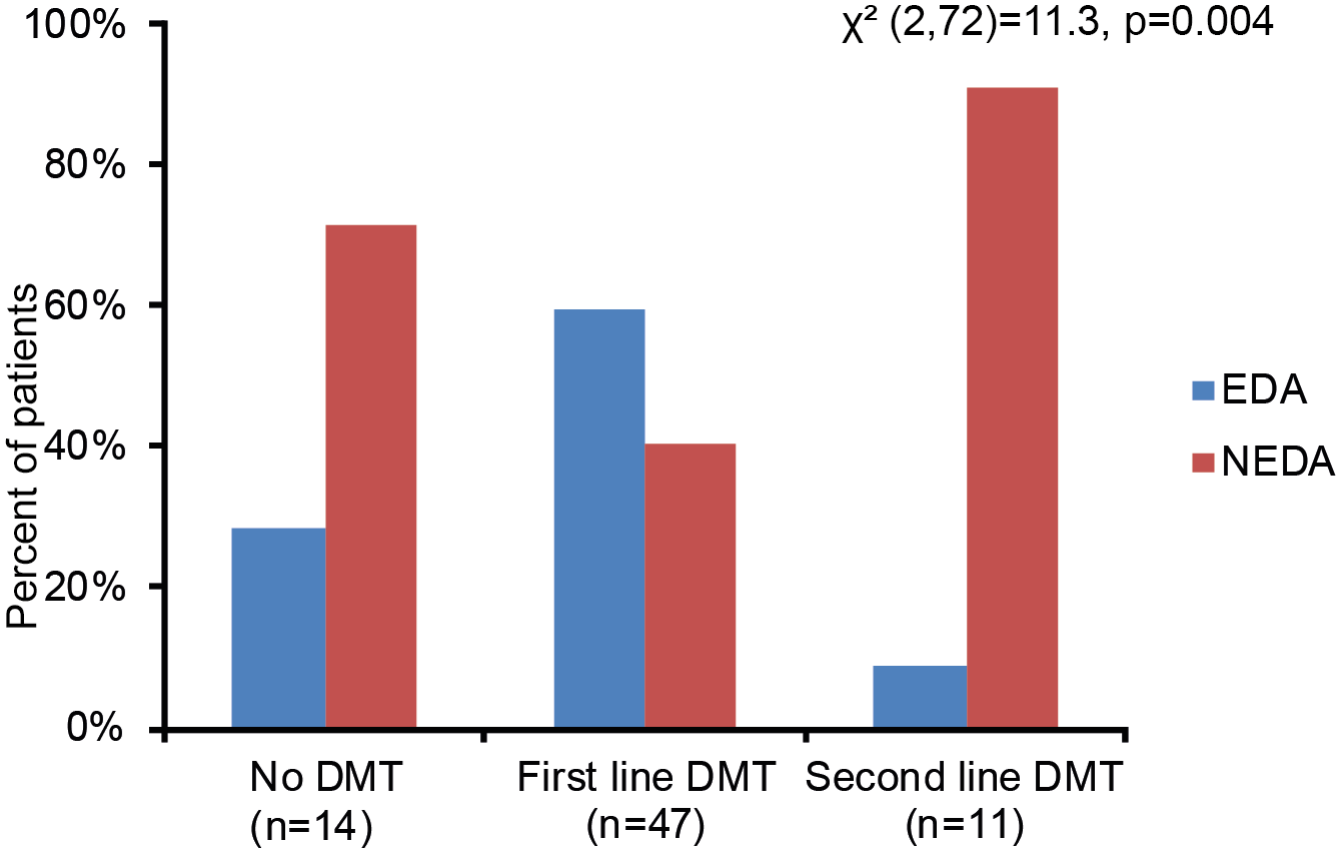
Disease activity in different treatment groups. Treatment groups as baseline of patients with EDA or NEDA one year later.

**Fig 5:**
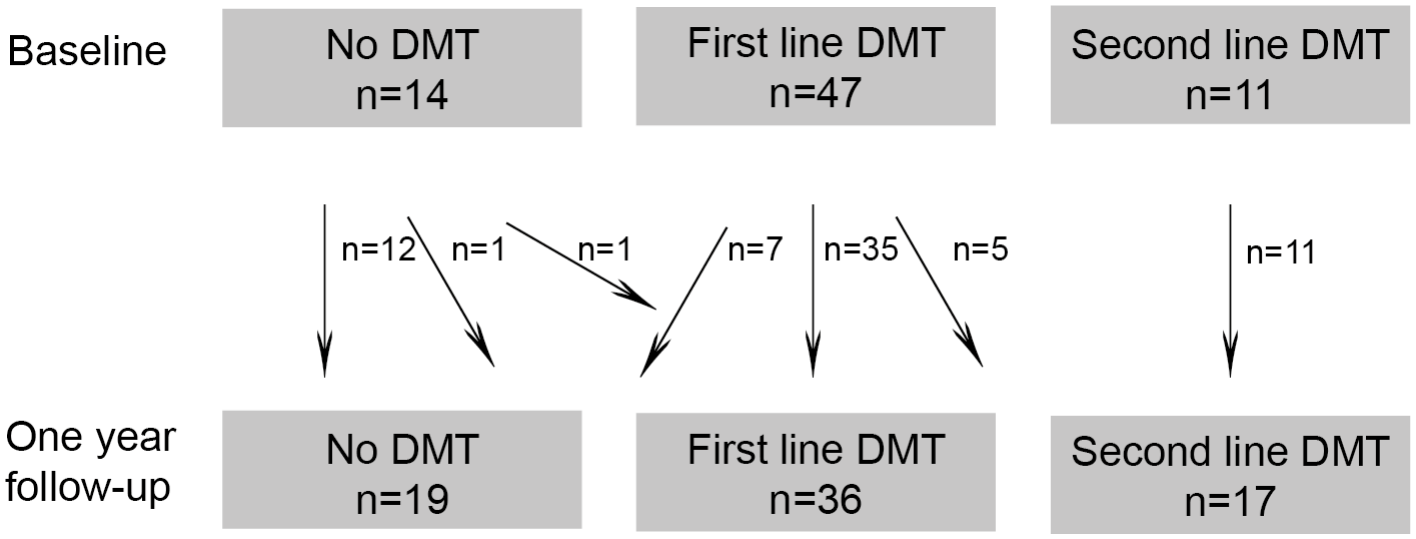
Patients in different treatment groups at baseline and follow-up. The arrows indicate change in treatment groups of patients from baseline to follow-up. Eight patients changed first line treatment during the period, not illustrated.

There were more patients using first line, and less patients using none or second line DMT at baseline in the EDA group compared to the NEDA group (χ^2^ (2, 72) = 11.3, p = 0.004)). Of the patients using no DMT at baseline, 29% (4/14) showed EDA one year later. Of the patients using first line DMT, 60% (28/47) showed EDA. Among patients using second line DMT at baseline, only 9% (1/11) were in the EDA group one year later (Fig 4). The patients on first line DMT had been using the same treatment for a mean of 10 months (SD 8.4) and the patients on second line DMT had been using the same DMT for a mean of 7 months (SD 4.6) at baseline. The NEDA patients had been using the same DMT for longer than the EDA patients (NEDA patients: mean 11.8 (SD 9.3) months, EDA patients: mean 7.0 (SD 5.2) months, p = 0.018). During the one year follow-up, 31% (22/72) of the patients changed DMT, either between first line treatments (n = 8) or between treatment groups (n = 14) (Fig 5). There were less patients changing DMT in the NEDA group than in the EDA group (NEDA patients with change in treatment: 18% (7/39), EDA patients with change in treatment: 45% (15/33), χ^2^ (1, 72) = 5.1, p = 0.023)).

**Table 2:** Disease characteristics, fatigue, depressive symptoms and cognitive assessment of patients according to evidence of disease activity at baseline and follow-up.

	**Baseline**		**Follow-up**	
	**EDA**	**NEDA**	**EDA**	**NEDA**
	**n = 33**	**n = 39**	**n = 33**	**n = 39**
**a. Disease characteristics**				
**Neurological disability, EDSS, mean (SD)**	1.8 (0.8)	2.0 (0.8)	2.2 (0.9)^1^	1.7 (0.6)^2^ ^,^ ^3^
**Disease duration, years, mean (SD)**	1.9 (1.8)	2.5 (2.1)	3.0 (1.8)	3.6 (2.1)
**Relapse rate, relapses/year, mean (SD)**	1.4 (0.8)	1.5 (1.8)	0.8 (0.5)^1^	0.7 (0.5)^2^
**T25FW, s, mean (SD)**	3.9 (0.5)	4.0 (0.6)	-	-
**9HP, s, mean (SD)**	21.0 (3.8)	20.3 (3.0)	-	-
	**Baseline**		**Follow-up**	
	**EDA**	**NEDA**	**EDA**	**NEDA**
	n = 32	n = 38	n = 32	n = 38
**b. Fatigue and depressive symptoms**				
**Fatigue, FSS, mean (SD)**	3.9 (1.7)	4.3 (1.8)	3.6 (1.7)	3.9 (2.0)
**Depressive symptoms, BDI, mean (SD)**	6.9 (5.1)	9.2 (6.3)	7.8 (6.4)	7.8 (5.7)
	**Baseline**		**Follow-up**	
	**EDA**	**NEDA**	**EDA**	**NEDA**
	n = 33	n = 38	n = 26	n = 30
**c. Cognitive assessment**				
**Processing speed, SDMT, mean (SD)**	54 (10)	52 (8)	55 (10)	54 (9)
**Processing speed, PASAT, mean (SD)**	47 (10)	45 (9)	-	-
**Verbal memory, CVLT, mean (SD)**	60 (11)	65 (10)	64 (9)^1^	68 (7)
**Visuospatial memory, BVMT-R, mean (SD)**	29 (6)	29 (5)	30 (4)	28 (5)

a. Disease duration: time from first symptom to baseline examinations, Relapse rate: total number of relapses/disease duration at baseline and follow-up, 9HP: 9 hole peg test, T25FW: timed 25 foot walk test. b. FSS: Fatigue Severity Scale, BDI: Beck Depression Inventory II. c. SDMT: Symbol Digit Modalities Test, PASAT: Paced Auditory Serial Addition Test 3 seconds, CVLT: California Verbal Learning Test, BVMT-R: Brief Visuospatial Memory Test Revised. Independent samples t-tests and paired samples t-tests as appropriate.

Bonferroni-corrected p-values<0.05 indicated with:

^1^difference between EDA at baseline and follow-up,

^2^difference between NEDA at baseline and follow-up,

^3^difference between EDA and NEDA at follow-up.

### Evidence of disease activity and fatigue, depressive symptoms and cognitive assessment

At baseline, EDA and NEDA patients had similar scores on fatigue, depressive symptoms and cognitive assessments (Table 2b and 2c). At one year follow-up, the EDA group showed an improvement in verbal memory (CVLT 60–64, Bonferroni-corrected p = 0.012). Further cognitive tests, fatigue and depression scores were stable in both groups (Table 2b and 2c).

### Evidence of disease activity and gray matter atrophy

Of the patients with available structural MRI data at follow-up, 51% (29/57) fulfilled the NEDA criteria. The patient groups and controls had similar intracranial volumes, ensuring comparability of GM volumes between the groups. Supratentorial WM volumes were similar. As illustrated in Table 3, there were differences in both cortical and subcortical GM volumes at baseline between the groups. Post-hoc pair-wise Bonferroni-corrected ANCOVA tests identified significant differences in cortical GM volume between NEDA and HC (F(1, 90) = 7.13, p = 0.027), while EDA and HC (F(1, 89) = 3.9, p = 0.153) and EDA and NEDA (F(1, 57) = 0.012, p = 0.912) cortical GM volumes were similar. Similar tests with subcortical GM volume as dependent variable revealed significant differences in subcortical GM volumes at baseline between NEDA and HC (F(1, 90) = 10.7, p = 0.006) and EDA and HC (F(1,89) = 8.1, p = 0.018), but not between NEDA and EDA (F(1, 57) = 0.4, p = 0.519). Thus both patient groups showed smaller subcortical volumes than HC at baseline, while cortical volumes of the EDA patients were not significant different from HC at baseline.

**Table 3:** Baseline MRI characteristics of patients and controls.

	NEDA		EDA		HC		ANCOVA		
	n = 29		n = 28		n = 61				
	Mean (mL)	SD	Mean (mL)	SD	Mean (mL)	SD	F	Partial η^2^	p
**Intracranial volume**	1540	121	1637	145	1617	126	0.927	0.016	0.399
**Supratentorial WM volume**	460	42	489	52	475	53	0.654	0.011	0.522
**Cortical GM volume**	473	35	494	44	500	35	4.21	0.069	0.017
**Subcortical GM volume**	56.7	4.8	59.0	3.9	60.7	4.4	8.14	0.126	<0.001

WM: white matter, GM: gray matter. The total neuroanatomical volumes, i.e. of both hemispheres combined, are presented. ANCOVAs were performed to test for differences in neuroanatomical volumes between the groups.

**Fig 6:**
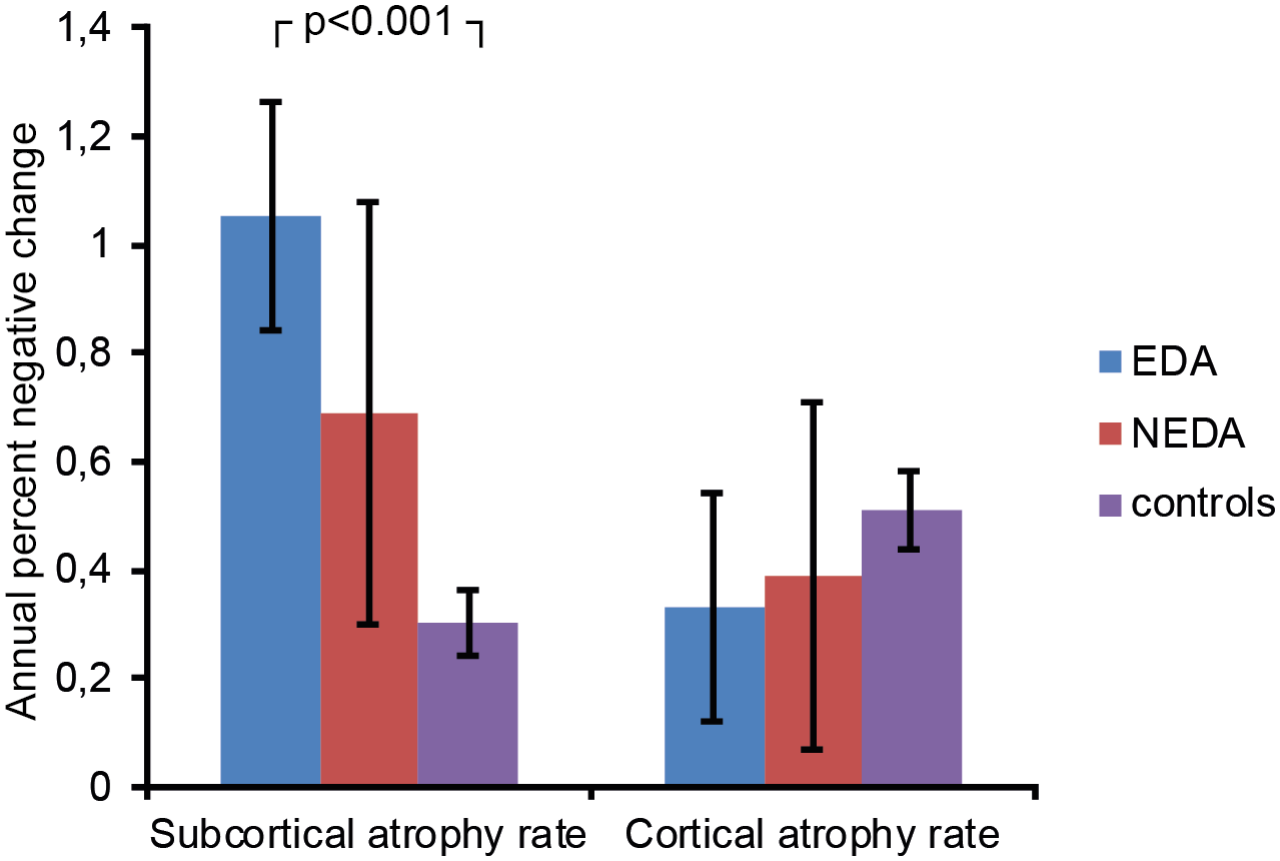
Annual gray matter atrophy rates. ANOVAs with Bonferroni-corrected post-hoc tests revealed that subcortical annual atrophy rates differed between patients with evidence of disease activity and healthy controls. Patients with no evidence of disease activity had similar atrophy rates as controls. Cortical atrophy rates were similar in all groups.

The annual percent change in subcortical GM volume differed between the groups (ANOVA: F(2, 118) = 8.1, p = 0.001). The atrophy rate was numerically higher in the EDA than the NEDA group, but this difference did not reach significance (EDA: -1.05%, NEDA: -0.68%, Bonferroni-corrected post-hoc test p = 0.305). EDA patients had higher subcortical atrophy rates than HC (Bonferroni-corrected post-hoc test p<0.001), while NEDA patients were not significantly different from HC (Bonferroni-corrected post-hoc test p = 0.130). The annual percent change in cortical GM volume was not significantly different between the groups (ANOVA: F(2, 118) = 0.201, p = 0.819) (Table 4, Fig 6).

**Table 4:** Annual percent change of MRI volumes of patients and controls.

	Annual percent change			
	RRMS	EDA	NEDA	HC
	n = 57	n = 28	n = 29	n = 61
**Intracranial volume, annual percent change, mean (SD)**	-0.11 (1.0)	-0.18 (1.28)	-0.04 (0.64)	-0.05 (0.18)
**Supratentorial WM volume, annual percent change, mean (SD)**	-0.26 (1.5)	-0.47 (1.7)	-0.06 (1.21)	0.08 (0.38)
**Cortical GM volume, annual percent change, mean (SD)**	-0.36 (1.9)	-0.33 (2.04)	-0.39 (1.72)	-0.51 (0.6)
**Subcortical GM volume, annual percent change, mean (SD)**	-0.86 (1.1)^1^	-1.05 (1.10)^2^	-0.68 (1.12)	-0.30 (0.43)

Annual percent change for patients and controls. Independent samples t-tests and ANOVAs with Bonferroni-corrected post-hoc tests were used to test for differences between the groups. Significant differences in atrophy rates were identified between:

^1^ RRMS and HC (p-value<0.001) and,

^2^ EDA and HC(p-value<0.001).

We further performed ANCOVAs with age and gender as covariates, group as a fixed factor and cortical and subcortical annual atrophy rates as dependent variables, which did not alter the result, neither for subcortical atrophy rate F(2, 118) = 7.2, p = 0.001) nor for cortical atrophy rate F(2, 118) = 0.122, p = 0.886).

## Discussion

We found evidence of disease activity after one year in almost half of the RRMS patients in this population-based cohort study. Disability at follow-up not only separated the two patient groups;-we also observed an *improvement* in disability in the NEDA group. Cognition was stable or improved in both patient groups, while only EDA patients had higher subcortical atrophy rates than HC.

An annual NEDA rate of approximately 50% is comparable to a recent cohort study, which found a one year NEDA rate in early MS patients of 0.46 [3]. Lower NEDA rates have been observed in most clinical trials, both for patients receiving DMTs and placebo [2,4,5], while a recent interim report on HDIT/HCT reported 78% NEDA after 3 years [6]. These difference may be caused by differences in inclusion criteria (in our study patients were included irrespective of disease activity, while most clinical trials include patients with active disease only), treatment (the patients in our cohort were assigned to treatment by their neurologist, not randomized) or disease duration (all patients in our cohort have disease duration ≤ 3 years). However, the present literature shows that we are still far from the goal of no evidence of disease activity in MS patients. The low proportion of NEDA among the patients receiving first line DMTs (40% (20/47)) is of particular interest. Even though there is some evidence that interferons delay the diagnosis in patients with clinically isolated syndrome [24], long term effects of first line DMTs in registry studies remain uncertain [25,26]. Our study supports that these drugs may not give sufficient protection against disease activity in early MS.

The NEDA patients improved in disability in our study, as in the recent HDIT/HCT study [6], an outcome which reported in MS studies [27]. Our findings may have been caused by a “regression to the mean”-effect in the NEDA patients, i.e. these patients might have an unusually active disease before study onset, and returned to a normal, and lower, disease activity during follow-up. However, baseline disease characteristics (relapse rate and EDSS) were similar between the patient groups. This supports our observation of disability improvement in the NEDA patients, which may reflect tissue repair in the absence of inflammation.

In the NEDA group, there was a trend towards an improvement in processing speed (probably the main cognitive domain affected in MS [28]) during the short observation period of this study, possibly as a consequence of disease stability. The EDA patients caught up with the NEDA group on verbal learning at follow-up, possibly due to a combination of practice effects and because they had not yet reached the ceiling of the test score at baseline. Patients in both groups had high levels of education and most were students or working [14], perhaps postponing, concealing or protecting them against cognitive decline [29,30]. Fatigue and depressive symptoms were also similar between the patient groups in our study, both at baseline and at follow-up, indicating that neither of these factors can predict EDA, nor are they the direct consequence of EDA in a one-year perspective.

In line with previous studies [10,14], the patients in our study showed both a thinner cerebral cortex and a smaller subcortical volume compared to controls, and annual subcortical GM atrophy rates were larger in patients than controls. The subcortical GM atrophy rates between the EDA and NEDA patients differed numerically, but were not significantly distinguishable in our sample. However, the subcortical atrophy rates of the patients with disease activity (EDA) were significantly higher than in the healthy controls. We therefore hypothesize that pathological neurodegeneration in this patient group drives the increased atrophy rates of the RRMS patients.

Pseudo-atrophy, the phenomenon that brain atrophy seems to accelerate with the onset of DMT in some MS patients, may obscure both clinical trials and observational studies, including the present study [31]. This effect may be strongest in the first months after DMT onset, and is suggested to be caused either by resolution of edema or a reduction in inflammatory cells, like microglia [32]. Gadolinium-enhancing lesions at trial onset has been linked to higher atrophy rates the first two years after natalizumab initiation, but not with disability progression, indicating that a reduction in inflammation causes benign and transient high atrophy rates [33]. Another study has found that pseudo-atrophy is most evident in WM, so that GM atrophy measures are still valid measures of true atrophy [34]. In our study most patients had been using the same DMT for more than half a year at baseline, so that at least some of the first critical time period of pseudo-atrophy had passed. The patients with evidence of disease activity more often changed treatment during the period. This change in treatment could have led to higher pseudo-atrophy rates in the EDA patient group. However, WM atrophy rates were similar between patients and controls, and between patients with and without evidence of disease activity. Thus the observed differences in GM atrophy rates in this study were most likely not caused by pseudoatrophy, but by true differences in volume loss.

It is still debated what is the most relevant outcome measures when following a RRMS population [1,21,35]. Scoring algorithms utilizing different combinations of disability, relapses and/or MRI assessments have been proposed, like the modified Rio score [36] and the Magnetic Resonance Disease Severity Score [37], in addition to “no evidence of disease activity” (NEDA). There are some obvious disadvantages to NEDA: The measure is dichotomous, so that a small asymptomatic WML gets the same weight as a major clinical relapse. Further, EDSS increase, relapses and WML are related, and a sum score like NEDA may just measure the same underlying pathology in many ways. And even though we do not find any substantial change in cognition in our one-year follow-up, there is considerable evidence that cognitive assessments should be included in clinical MS evaluations [7]. However, NEDA does not add any extra examinations to the standard clinical evaluation of MS patients, it fits with international treatment guidelines [12,38], and the ambition intrinsic to the term may keep clinicians alert and ensure individualized treatment of each MS patient.

Our population-based patient sample allowed us to study the disease development in a real-world sample of early RRMS patients. The access to almost complete clinical information at one year follow-up, the well-matched healthy controls and the stability of MRI acquisition throughout the study were the strengths of our study.

A limitation of this study was the short follow-up time. Some of the eligible patients in the region declined to participate, possibly leading to a somewhat biased patient sample. We further lost some patients when doing structural MRI follow-up. Even though the patients that were not included in the follow-up were similar to the whole RRMS sample demographically and clinically, we cannot rule out a loss-to-follow-up bias. Furthermore, our sample size was modest, and larger samples might reveal more group differences. The long scan interval in HC compared to the patient groups (3.5 vs 1.1 years) resulted in older HC at follow-up compared to the patients, so that our result could partly have resulted from the age difference between the groups. However, the age of the participants at the mid-time between the two MRI acquisitions was similar, so that the atrophy measurements span similar age levels in both groups. The longer time interval may also have allowed for more differences in MRI acquisitions between the scan periods, increasing the risk of a measurement error in the HC group. White matter lesions may interfere with automatic brain segmentations, and in this study, lesions masks were not available for lesion filling. This may have lead to an underestimation of gray matter volume and atrophy rates observed, especially in patients with new juxtacortical or infratentorial lesions [39]. However, the impact of lesion filling on longitudinal analyses is not known [40]. Further, the within-subject approach applied to the longitudinal analyses in this study ensures estimation of precise intraindividual atrophy rates [22]. Thus, our results should be validated in larger patient and control samples with longer follow-up time.

## Conclusions

The striking differences in EDSS development at one year follow-up, combined with the high subcortical atrophy rates in EDA patients compared to controls, support the use of NEDA as an outcome measure in MS. The high subcortical atrophy rates in the EDA patients, combined with the high proportion of patients treated with first line DMTs in this patient group, underlines the need for treatment strategies targeting GM atrophy in early RRMS, especially in patients with evidence of disease activity.

## Supporting Information

S1 Table Demographic information of RRMS patients with clinical and MRI information. (DOCX)

S2 Table Disease characteristics, treatment, disease activity, fatigue, depressive symptoms and cognitive assessment of RRMS patients with clinical and MRI information at baseline and follow-up. (DOCX)
