# Supplementary material for: A Longitudinal Study of Disability, Cognition and Gray Matter Atrophy in Early Multiple Sclerosis Patients According to Evidence of Disease Activity
Source: PLoS One. 2015 Aug 17;10(8):e0135974. doi: 10.1371/journal.pone.0135974 (PMC4539191; doi:10.1371/journal.pone.0135974)
Supplement: S1 Table — (DOCX) [file pone.0135974.s001.docx]

**SI Table 1. Demographic information of RRMS patients with clinical and MRI information**

|  | **RRMS**  **Clinical** | **RRMS**  **MRI** |
| --- | --- | --- |
|  | **(n=72)** | **(n=57)** |
| **Female, n (%)** | 52 (72) | 40 (70) |
| **Age, years, mean (SD)** | 34.3 (7.0) | 34.6 (7.2) |
| **Education, years, mean (SD)** | 15.1 (2.3) | 15.1 (2.1) |
| **Follow-up period, months, mean (SD)** | 13.4 (2.3) | 13.5 (1.6) |

**SI Table 1. legend:** RRMS Clinical: RRMS patients with clinical information at baseline and follow-up, RRMS MRI: RRMS patients with complete structural MRI at baseline and follow-up**.** Age: age at baseline examinations, Education: years of education at baseline examinations, Follow-up period: time between MRIs or journal information at baseline and follow-up.
