# Supplementary material for: A Longitudinal Study of Disability, Cognition and Gray Matter Atrophy in Early Multiple Sclerosis Patients According to Evidence of Disease Activity
Source: PLoS One. 2015 Aug 17;10(8):e0135974. doi: 10.1371/journal.pone.0135974 (PMC4539191; doi:10.1371/journal.pone.0135974)
Supplement: S2 Table — (DOCX) [file pone.0135974.s002.docx]

**SI Table 2. Disease characteristics, treatment, disease activity, fatigue, depressive symptoms and cognitive assessment of RRMS patients with clinical and MRI information at baseline and follow-up.**

|  | **Baseline** | | | **Follow-up** | | |
| --- | --- | --- | --- | --- | --- | --- |
| **a) Disease characteristics** | **RRMS**  **Clinical** | **RRMS**  **MRI** | | **RRMS Clinical** | **RRMS**  **MRI** | |
|  | n=72**^1^** | n=57 | | n=72 | n=57 | |
| **Neurological disability, EDSS** | 1.9 (0.8) | 1.9 (0.8) | | 1.9 (0.8) | 1.9 (0.8) | |
| **Disease duration, years** | 2.2 (2.0) | 2.3 (2.1) | | 3.4 (2.0) | 3.5 (2.1) | |
| **Relapse rate, relapses/year** | 1.5 (1.4) | 1.5 (1.4) | | 0.7 (0.5) | 0.7 (0.4) | |
| **25 FWT, s** | 3.9 (0.6) | 3.9 (0.5) | | - | - | |
| **9HP, s** | 20.6 (3.4) | 20.5 (3.4) | | - | - | |
|  | **Baseline** | | | **Follow-up** | | |
| b) Disease Modulatory Treatment | **RRMS**  **Clinical** | **RRMS**  **MRI** | | **RRMS Clinical** | **RRMS**  **MRI** | |
|  | n=72 | n=57 | | n=72 | n=57 | |
| **None, n (%)**  **First line, n (%)**  **Second line, n (%)** | 14 (19)  47 (67)  11 (14) | 11 (19)  36 (63)  10 (18) | | 19 (26)  36 (50)  17 (24) | 14 (25)  27 (47)  16 (28) | |
| **Change in DMT, n (% of total)** | - | - | | 22 (31) | 18 (32) | |
|  | **Baseline** | | | **Follow-up** | | |
| c) Evidence of disease activity | **RRMS**  **Clinical** | | **RRMS**  **MRI** | **RRMS Clinical** | | **RRMS**  **MRI** |
|  | n=72 | | n=57 | n=72**^3^** | | n=57 |
| **EDA, n (%)** | - | | - | 33 (46) | | 28 (49) |
| **Disability progression, EDSS increase ≥1, n (%)** | - | | - | 11 (15) | | 10 (18) |
| **New relapse, n (%)** | - | | - | 10 (14) | | 7 (12) |
| **Radiological progression n, (%)** | - | | - | 17 (27) | | 15 (26) |
|  | **Baseline** | | | **Follow-up** | | |
| d) Fatigue and depressive symptoms | **RRMS**  **Clinical** | | **RRMS**  **MRI** | **RRMS Clinical** | | **RRMS**  **MRI** |
|  | n=72**^1^** | | n=57^2^ | n=72**^3^** | | n=57**^4^** |
| **Fatigue, FSS** | 4.1 (1.7) | | 4.1 (1.8) | 3.8 (1.9) | | 3.8 (1.9) |
| **Depressive symptoms, BDI** | 8.1 (5.9) | | 8.1 (5.9) | 7.8 (6.0) | | 8.1 (6.0) |
|  | **Baseline** | | | **Follow-up** | | |
| e) Cognitive assessment | **RRMS**  **Clinical** | | **RRMS**  **MRI** | **RRMS Clinical** | | **RRMS**  **MRI** |
|  | n=72**^1^** | | n=57 | n=72**^3^** | | n=57**^4^** |
| **Processing speed, SDMT, sum of correct answers** | 53.1 (9.1) | | 53.1 (8.8) | - | | 54.9 (8.9) |
| **Processing speed, PASAT, sum of correct answers** | 46.4 (9.5) | | 46.8 (9.4) | - | | - |
| **Verbal memory, CVLT, sum of five trials** | 62.3 (10.4) | | 62.2 (10.1) | - | | 66.1 (8.3) |
| **Visuospatial memory, BVMT-R, sum of three trials** | 28.5 (5.5) | | 28.4 (5.4) | - | | 29.0 (4.6) |

**Table 2 footnotes:** ^a^ n=71 for disease characteristics and cognitive assessment except EDSS, and n=70 for questionnaires, ^b^ n=56 for questionnaires, ^c^ n=62 for MRI evaluation of new lesions, n=56 for cognitive assessment and n=53 for questionnaires, ^d^ n=51 for cognitive assessment and n=50 for questionnaires.

**Table 2 legend:** Data are presented as number (%: percentage) or mean (SD: standard deviation) as appropriate. RRMS: relapsing-remitting multiple sclerosis, RRMS Clinical: RRMS patients with clinical information at baseline and follow-up, RRMS MRI: RRMS patients with complete structural MRI at baseline and follow-up.

1. Disease duration: time from first symptom to baseline examinations, Relapse rate: total number of relapses/disease duration at baseline and follow-up, 9HP: 9 hole peg test of dominant hand, T25FW: timed 25 foot walking test.
2. First line: glatiramer acetate or interferons, Second line: natalizumab or fingolimod. Change in DMT: patients who changed DMT between baseline and follow-up examinations.
3. EDA: patients with evidence of disease activity, Disability progression: patients with increase in EDSS≥1, New relapse, patients with new neurological symptoms not associated with fever or infection lasting for at least 24 hours and accompanied by new neurological signs, radiological progression: patients with new or enlarging T2 or FLAIR lesion or new T1 Gadolinium enhancing lesion at follow-up compared to baseline scans.
4. FSS: Fatigue Severity Scale, BDI: Beck Depression Inventory II
5. SDMT: Symbol Digits Modalities Test, PASAT, Paced Auditory Serial Addition Test 3 seconds, CVLT: California Verbal Learning Test (original word list used at baseline and alternate word list used at follow-up), BVMT-R: Brief Visuospatial Memory Test Revised (different forms used at baseline and follow-up).
